# Retraction Note: Mesenchymal stem cell-derived exosomal miR-146a reverses diabetic β-cell dedifferentiation

**DOI:** 10.1186/s13287-022-03184-5

**Published:** 2022-10-08

**Authors:** Qin He, Jia Song, Chen Cui, Jinbang Wang, Huiqing Hu, Xinghong Guo, Mengmeng Yang, Lingshu Wang, Fei Yan, Kai Liang, Zhaojian Liu, Fuqiang Liu, Zheng Sun, Ming Dong, Xinguo Hou, Li Chen

**Affiliations:** 1 Department of Endocrinology, Qilu Hospital, Cheeloo College of Medicine, Shandong University, No. 107 Wenhua Xi Road, Jinan, 250012 Shandong China; 2 Department of Cell Biology, Cheeloo College of Medicine, Shandong University, Jinan, 250012 China; 3 Institute of Endocrine and Metabolic Diseases of Shandong University, Jinan, 250012 China; 4 Key Laboratory of Endocrine and Metabolic Diseases, Shandong Province Medicine & Health, Jinan, 250012 China; 5 Jinan Clinical Research Center for Endocrine and Metabolic Diseases, Jinan, 250012 China

## Retraction Note to: Stem Cell Research & Therapy (2021) 12:449 https://doi.org/10.1186/s13287-021-02371-0

The Editors-in-Chief have retracted this article because there are regions of overlap between Fig. 2A and Fig. 4A. The Editors-in-Chief therefore no longer have confidence in the data presented.

Li Chen does not agree to this retraction. None of the other authors has responded to any correspondence from the publisher about this retraction.

